# Cellular Response to Surface Morphology: Electrospinning and Computational Modeling

**DOI:** 10.3389/fbioe.2018.00155

**Published:** 2018-10-24

**Authors:** Anna Denchai, Daniele Tartarini, Elisa Mele

**Affiliations:** ^1^Department of Materials, Loughborough University, Loughborough, United Kingdom; ^2^Department of Civil Engineering, University of Sheffield, Sheffield, United Kingdom

**Keywords:** bio-interfaces, surface topography, electrospinning, micro-patterning, mathematical modeling

## Abstract

Surface properties of biomaterials, such as chemistry and morphology, have a major role in modulating cellular behavior and therefore impact on the development of high-performance devices for biomedical applications, such as scaffolds for tissue engineering and systems for drug delivery. Opportunely-designed micro- and nanostructures provides a unique way of controlling cell-biomaterial interaction. This mini-review discusses the current research on the use of electrospinning (extrusion of polymer nanofibers upon the application of an electric field) as effective technique to fabricate patterns of micro- and nano-scale resolution, and the corresponding biological studies. The focus is on the effect of morphological cues, including fiber alignment, porosity and surface roughness of electrospun mats, to direct cell migration and to influence cell adhesion, differentiation and proliferation. Experimental studies are combined with computational models that predict and correlate the surface composition of a biomaterial with the response of cells in contact with it. The use of predictive models can facilitate the rational design of new bio-interfaces.

## Introduction

The natural regeneration process of human tissues is strongly regulated by the interaction of cells with the extracellular matrix (ECM) (Lutolf and Hubbell, 2005; Liu and Wang, 2014). ECM is a dynamic and complex fibrous network of proteins and polysaccharides, such as collagen, elastin, fibronectin, laminin, proteoglycans and glycosaminoglycans. Cells interact with ECM by transmembrane receptors, known as integrins, that ligate with specific motifs of ECM proteins, for example arginine, glycine and arginylglycylaspartic acid (RGD) peptides (Anderson et al., 2016; Dalby et al., 2018). Cells continuously remodel the ECM environment, which, in turn, influences cell behavior and fate (differentiation, proliferation and migration) by biochemical, physical and mechanical signals (Geiger et al., 2001), and provides structural support to cells. Recent studies have investigated the effects of ECM physical properties, particularly porosity, topography and hierarchical 3D architecture, on cellular functions, and extrapolated rules to design structures for effective tissue regeneration (Li et al., 2017; Marino et al., 2017; Lin et al., 2018).

One of the technologies that is widely used to produce ECM-mimicking structures and particularly to replicate the fibrillar architecture of ECM is electrospinning (Khorshidi et al., 2016). The electrospinning technique allows the production of networks of fibers with a diameter in the range of few nm to few μm via the application of electrical forces to polymer solutions or melts (Bhardwaj and Kundu, 2010; Mele, 2016; Zhang et al., 2016). Structural modifications of electrospun nanofibres, such as altering topographical characteristics and inducing porosity, can be achieved by controlling and varying the process parameters (polymer concentration, applied voltage, evaporation rate of the solvent used). Similarly, changes to the final makeup of the fibrous network, such as alignment and patterning of fibers, can be obtained by modifications of the electrospinning apparatus or post-processing.

This mini review analyses a selection of recent works on the use of solution electrospinning to create nanofibres with engineered surface topography (random, aligned and patterned fibers) for controlling adhesion, differentiation, and migration of different cells lines. The mini review is divided in two main sections: the first one will focus on experimental studies on electrospun fibers that provide physical cues for cell growth and differentiation; the second section will discuss computational models to predict cell behavior on micropatterns. Although mathematical models that simulate cell behavior on electrospun fibers are not currently available, the computational approaches here discussed can be adapted, in the future, to electrospun scaffolds and used to elucidate the underlying mechanisms responsible for cell-fiber interaction.

## Effects of FIBER topography and micro-patterning on cellular response

Multiple studies have demonstrated that the morphology and roughness of fibers produced by electrospinning influence cell adhesion, proliferation, and orientation (Sill and von Recum, 2008; Xie et al., 2008; Bergmeister et al., 2013; Cirillo et al., 2014; Zhu et al., 2015; Sun et al., 2018). All factors that are imperative for successful tissue regeneration (Agarwal et al., 2008). Cells can sense topographical structures on a surface by filipodia that are actin-rich protrusions (0.1–0.3 μm in diameter) of the cell membrane and are involved in cell contact guidance (Mattila and Lappalainen, 2008; Dalby et al., 2014). If nanoscale aligned features are present onto a surface, filopodia tend to orient along the direction of the features and determine cytoskeleton orientation. Focal adhesions at the cell membrane mediate the initial cell-biomaterial interaction, with integrin ligands in direct contact with the substrate and connected to the actin micro-filaments of the cell cytoskeleton by a 40-nm stratum, which includes focal adhesion kinase (FAK), paxillin, talin, and vinculin (Kanchanawong et al., 2010).

This section of the review will discuss how electrospun mats with controlled porosity and surface morphology have been used to influence the behavior of mesenchymal stem cells (MSCs) (Jiang et al., 2015; Yin et al., 2015; Baudequin et al., 2017; Lin et al., 2017; Liu et al., 2017; Nedjari et al., 2017; Su et al., 2017; Zhang et al., 2017; Ghosh et al., 2018; Jin et al., 2018; Rahman et al., 2018; Sankar et al., 2018) and human umbilical vein endothelial cells (HUVECs) (Fioretta et al., 2014; Xu et al., 2015; Shin et al., 2017; Taskin et al., 2017; Yan et al., 2017; Ahmed et al., 2018). The literature on other cell lines, such as on myoblasts (Mele et al., 2015; Jun et al., 2016; Park et al., 2016; Tallawi et al., 2016; Abarzúa-Illanes et al., 2017; Yang et al., 2017) and neuron-like cells (Binan et al., 2014; Xie et al., 2014; Malkoc et al., 2015; Xue et al., 2017; Hajiali et al., 2018; Xia and Xia, 2018), will not be analyzed in detail here but a summary of it is reported in Table 1.

**Table 1:** Summary of the recent literature on the use of electrospun fibers to control morphology, alignment and differentiation of diverse cell lines.

**Cells**	**Material**	**Fiber characteristics**	**Main outcomes**	**References**
Human MSCs	Poly (ε-caprolactone)	Randomly distributed fibers; Diameter: ~ 630 nm; Surface roughness: ~ 2 μm.	Recruitment of MSCs *in vivo* and *ex vivo*; Recruitment of macrophages *in vivo*; Phenotype transition of adhered macrophages from pro-inflammatory (M1) to pro-healing (M2).	Zhang et al., 2017
Human MSCs	Poly (ε-caprolactone); Poly (ε-caprolactone)-gelatine	Randomly distributed and aligned fibers; Diameter: 600–780 nm; Porosity: 78–86%.	Cardiomyogenesis; Cytoskeletal arrangement; Changes in the cellular and nuclear morphology.	Ghosh et al., 2018
Human MSCs	Poly (L-lactic acid)	Randomly distributed and aligned fibers coated with poly (3,4-ethylenedioxythiophene; Diameter: ~ 950 nm.	Synergic effect of fiber alignment and electrical stimulation; Promotion of cellular activity and proliferation.	Jin et al., 2018
Human adipose-derived MSCs	Poly (L-lactide ε-caprolactone) and fibrinogen	Random and aligned fibers; Diameter: 200–500 nm; Patterning of electrospun mats using honeycomb shaped collector produced by photolithography; Honeycomb: 160 μm internal diameter, walls of 20 μm width and 60 μm height.	Homotypic interaction of MSCs on honeycomb scaffolds; Osteogenic differentiation of MSCs on honeycomb scaffolds.	Nedjari et al., 2017
Human adipose-derived MSCs	SU-8 photoresist	Randomly oriented fibers; Diameter: 550 nm; Patterning of electrospun mats by photolithography; Pattern dimensions: 20 μm ridges, 20 μm grooves, 5 μm pattern height.	Orientation and alignment of cells resembling the *in vivo* anisotropic multilamellar architecture of bone; Osteodifferentiation of MSCs.	Sankar et al., 2018
Human bone marrow MSCs	Poly (ε-caprolactone)	Random-aligned-random structure; Diameter: 240–450 nm.	Regional induction of MSCs toward tenogenesis and osteogenesis; Collagen deposition.	Lin et al., 2017
Human dental pulp MSCs	Polystyrene	Randomly distributed fibers; Diameter: 300–500 nm; Surface roughness: 0.8 μm.	Increased the expression of bone morphogenetic proteins and Wnt ligands; Odontoblast differentiation of MSCs; Dentin regeneration.	Rahman et al., 2018
Mouse MSCs (C3H10T1/2)	Poly (L-lactic acid)	Random and aligned fibers; Diameter: 740–1070 nm.	Up-regulation of tendon-specific markers for MSCs on aligned fibers; Tendon-like tissue regeneration *in vivo* for aligned fibers; Bone formation *in vivo* for random fibers.	Yin et al., 2015
Mouse MSCs (C3H10T1/2)	Polylactic acid and polycaprolactone	Random and aligned coaxial fibers; Diameter: ~ 2 μm; Porosity: 82–84%.	Expression of tendon-related markers; Tenogenic differentiation of mouse MSCs.	Baudequin et al., 2017
Rat bone marrow MSCs	Poly (ε-caprolactone) and poly (ethylene glycol); Chitosan	Random and aligned fibers; Diameter: 200–600 nm; 3D multi-layered scaffolds: layers of fibers within a porous chitosan matrix.	Ligamentogenesis and partially decreased osteogenesis for MSCs for aligned nanofibers embedded scaffolds *in vitro*; Regeneration of periodontal ligament *in vivo* for aligned nanofibers embedded scaffolds; High expression levels of periostin and formation of tooth-supporting mineralised tissue in the regenerated periodontium for aligned scaffolds.	Jiang et al., 2015
Rat bone marrow MSCs	Poly (ε-caprolactone)	Random and aligned fibers; Diameter: 820–1000 nm; Application of mechanical tension-stress after cell seeding.	Osteogenic differentiation of MSCs onto aligned fibers; Expression of osteogenic genes on aligned fibers; enhanced expression of osteogenic genes after mechanical stimulation.	Liu et al., 2017
Rat adipose-derived MSCs	Poly (ε-caprolactone)	Random and aligned fibers; Diameter: 1 μm; Patterning of electrospun mats using copper mesh with grid length of 830 μm as collector.	Upregulated levels of anti-inflammatory and pro-angiogenic cytokines *in vitro* for MSCs on patterned mats; Therapeutic effects of the fibers in a skin excisional healing model *in vivo*.	Su et al., 2017
HUVECs	Poly (D,L-lactide) and polycaprolactone	Random and aligned fibers; Diameter: 500–700 nm; Patterning of electrospun mats using a wire spring with interval distances of 300, 800, and 1500 μm as collector.	Modification of cytoskeleton morphology; Cell alignment and polarization on aligned fibers; Expression of angiogenesis-related genes.	Xu et al., 2015
HUVECs	Polycaprolactone and polyethyleneoxide	Nanostructured, random fibers. Diameter: 4–20 μm.	Enhanced cells' proliferation; Stimulation of adhesion complex formation on nanotextured fibers.	Taskin et al., 2017
HUVECs	Poly (L-lactide)	Random and aligned fibers; Patterning of electrospun mats by femtosecond laser ablation; Pattern dimensions: grooves distance of 20.9 and 81.3 μm; grooves width of 9.4 and 7.6 μm; grooves depth of 12.5 and 13.9 μm.	Changes in morphology and orientation of cells on micropatterned scaffolds; Reduction of monocytes adhesion on the micropatterned mats; Anti-inflammatory response.	Shin et al., 2017
HUVECs	Poly (L-lactic acid)	Random fibers; Diameter: 540 nm; Patterning of electrospun mats by hot embossing; Pattern dimensions: 50, 100, and 200 μm wide grooves.	Cells alignment along the direction of the grooves; Expression of endothelial biomarkers by cells cultured on micropatterned scaffolds.	Yan et al., 2017
HUVECs	Poly (lactic-co-glycolic acid)	Aligned fibers; Diameter: 0.5–10 μm.	Cell alignment and polarization on fibers with intermediate diameter; Stimulation of a migratory phenotype.	Ahmed et al., 2018
C2C12 myoblasts and neonatal rat cardiomyocytes	Poly (glycerol sebacate) and poly (caprolactone)	Random fibers; Diameter: 1.2 μm; Patterning of electrospun mats using a microstructured collector; Parallel grooves of 10 μm diameter and interspatial distances of 200 and 7 μm; Square-shaped structures of 100 μm size and 50 μm distance. Surface roughness: 0.4–1.3 μm.	Cells alignment along parallel grooves topography.	Tallawi et al., 2016
C2C12 myoblasts	Poly (caprolactone)	Random and aligned fibers; Diameter: 0.8-2.5 μm. Distance between aligned fibers: 2.2 and 13.8 μm.	Uniaxial orientation and elongation of cells on aligned fibers; Myogenic differentiation and elongation of myotubes along the aligned fibers.	Park et al., 2016
C2C12 myoblasts	Poly (L-lactic acid)	Random fibers; Diameter: 720 nm; Patterning of electrospun mats using a femtosecond laser ablation; Parallel grooves of 5 μm width and spacing of 10, 25, and 80 μm.	Cells alignment along the micro-grooves; Regulation of cellular adhesive morphology, proliferation, and distribution of focal adhesion proteins.	Jun et al., 2016
C2C12 myoblasts	Poly (ε-caprolactone) and poly (lactic-co-glycolic acid)	Random and aligned fibers; Diameter: 0.4–3.2 μm;	Increased alignment and aspect ratio of myotubes on aligned fibers.	Abarzúa-Illanes et al., 2017
Neuron-like PC12 cells	Poly (caprolactone) and gelatin; Collagen; Polystyrene	Random fibers; Diameter: 440 nm; Patterning of electrospun mats using polystyrene 5 μm wide grooves and 18 μm diameter wells by thermal fusion.	Increased extension of neurites within the grooves; High neurite length per differentiated cell for the micropatterned substrates.	Malkoc et al., 2015
Neural stem cells	Polyphenylene sulfone	Random and aligned fibers; Diameter: 735 nm.	Enhanced neuronal differentiation on the fibrous scaffolds; Growth and activity of primary neural cells on nanofibres; Parallel axon growth on aligned nanofibers.	Hajiali et al., 2018

### Mesenchymal stem cells

MSCs are multipotent stem cells that are primarily isolated from bone marrow, but they can also be found in adipose tissue, dental pulp, placenta, umbilical cord and other vascularized tissues throughout the body (Lv et al., 2014; Tartarini and Mele, 2015). MSCs are of great interest in regenerative medicine, because of their therapeutic effects, such as: ability to differentiate into various cell types and therefore promote regeneration of a wide range of tissues (bone, cartilage, muscle, marrow, tendon, ligament, nervous tissue, and skin); secretion of bioactive molecules for tissue repair; migration to inflamed tissues and modulation of local inflammation; immunomodulatory functions (Sharma et al., 2014).

In a recent research, Zhang et al. have studied how the topography and fibrillar organization of electrospun poly (ε-caprolactone) (PCL) fibers influences the recruitment of MSCs *in vivo* and *ex vivo* (Zhang et al., 2017). PCL mats (randomly distributed fibers) were implanted into the subcutaneous tissue of rats and the results were compared with solid PCL films (not electrospun). It was observed that, during the initial post-implantation period (1 day), a great number of macrophages with M1 phenotype (pro-inflammatory) were recruited to the PCL fibers, differently from solid PCL. This was attributed to the high surface area of the fibers and the porosity of the electrospun mats that promoted protein adsorption from the surrounding tissue, such as complement C3a (a chemo-attractant responsible to activate and recruit immune cells), fibronectin and vitronectin. After 4 and 7 days of implantation, the PCL fibers attracted host MSCs and modulated macrophages polarization with an increased number of cells exhibiting M2 (pro-healing) phenotype. While the number of M2 cells continuously increased over the entire period of implantation for PCL fibers, this was not the case for solid PCL where a large population of M1 cells was retained. Migration of MSCs was also observed in *ex vivo* experiments conducted with the implanted PCL samples. It was found that the macrophages at the implanted PCL mats secreted high levels of SDF-1, a chemokine that mediates MSCs recruitment by interacting with CXC chemokine receptors on the MSCs membrane. The study concluded that the physical organization of the PCL electrospun network induced the phenotype M1-to-M2 transition of macrophages that attracted MSCs at the implantation site by releasing SDF-1. This cascade of events was beneficial to stimulate tissue repair. PCL electrospun fibers have been used also to stimulate the production of pro-angiogenic and anti-inflammatory paracrine factors in rat adipose-derived MSCs (Ad-MSCs) (Su et al., 2017) and in skin excisional wound-healing model in rats (Table 2). Ad-MSCs were seeded on three types of electrospun PCL fibers, random (REF), aligned (AEF) and with a mesh pattern (MEF). It was observed that scaffolds with oriented fibers (AEF and MEF) promoted the expression of PGE2 (Prostaglandin E2, a potent inflammatory mediator), iNOS (inducible Nitric Oxide Synthase), VEGF (vascular endothelial growth factor) and HGF (hepatocyte growth factor), compared to REF scaffolds. In order to elucidate the molecular signaling mechanism responsible for the paracrine secretion of Ad-MSCs, the cells were treated with an inhibitor of NF-kB (a transcription factor that induces the expression of pro-inflammatory genes) and this significantly reversed the paracrine response of MSCs to the electrospun scaffolds. The authors therefore speculated that, in the presence of the scaffolds, MSCs behaved as if they were exposed to an external inflammatory stimulus. Similar results have been recently reported for MSCs cultured on electrospun fibers of PCL/polytetrahydrofuran (PTHF) urethane (P fibers) and PCL-PTHF urethane/collagen I (PC fibers) (Jiang et al., 2018). In this case, down-regulation of genes that contribute to inflammation and suppression of the NF-kB pathway signaling pathway were achieved by changing the mechanical properties of the fibers. PC fibers with a Young's modulus of 4.3 MPa were able to suppress inflammation, differently from P fibers (Young's modulus of 6.8 MPa).

**Table 2:** Summary of main results reported in selected recent papers on electrospun scaffolds used *in vivo* experiments.

**Scaffolds**	***In vivo* outcomes**	**References**
Mono-component (MC) and bi-component (BC) conduits made of random PCL and PCL/gelatin fibers, respectively, implanted in rat sciatic nerve defects.	Formation of numerous myelinated axons and vasculature in the MC conduit group; fibrous tissue and inflammatory cells with no evidence of myelinated axons for BC conduits, due to gelatin degradation or mechanical collapse. Superior functional recovery recorded for MC conduits over BC conduits after 18 weeks of implantation. Recover of tibialis anterior and gastrocnemius muscle weights after 18 weeks for MC conduit group; muscle atrophy for BC conduit group.	Cirillo et al., 2014
Random and aligned PCL-PEG fibers within a chitosan matrix implanted in a surgically created defect in maxillary first molar of rats.	Rat bone marrow mesenchymal stem cells (rBMSCs) with spindle shape and oriented actin filaments on scaffolds consisting of aligned fibers; while rBMSCs with polygonal or dentritic shape of scaffolds with random fibers. Increased ligamentogenesis and partially decreased osteogenesis for rBMSCs for scaffolds with aligned fibers. Increased stability and maturation of the periodontal ligament matrix, and increased regenerated bone volume and density for scaffolds with aligned fibers.	Jiang et al., 2015
Vascular grafts with oriented PCL microfibers coated with electrospun random PCL nanofibres and implanted in rat abdominal aorta.	Enhanced growth of vascular smooth muscle cells (VSMCs) after 2 and 4 weeks of implantation. Regeneration of arteries with notable VSMCSs vaso-activity after 12 weeks of implantation, and synthesis of elastin and collagen type I/II with phenotypic and structural similarities to the native arteries. Complete endothelialisation after 4 weeks with endothelial cells (ECs) having a morphology similar to the native endothelium. Regeneration of healthy and functional neaoarteries where VSMCs and ECs response to the endothelial-specific activator acetylcholine, hence showing vasodilation.	Zhu et al., 2015
Random PCL fibers implanted into the subcutaneous tissues of rats.	Macrophage recruitment, elongation and increased the expression of Arginase-1 or IL-4. Macrophage phenotype transition from M1 (pro-inflammatory) to M2 (pro-healing). High adsorption of proteins, particularly the chemotactic factor Complement C3a, vitronectin and fibronectin. Macrophages' secretion of high levels of SDF-1, a chemokine that mediates MSCs recruitment by interacting with CXC chemokine receptors on the MSCs membrane.	Zhang et al., 2017
Conditioned-medium (CM) from Ad-MSCs cultured on oriented (AEF and MEF) PCL fibers. CM applied to a skin wound-healing model.	High wound closure rate for animals treated with the MSC-MEF CM. Collagen deposition in a fine reticular pattern for group of MSC-MEF CM. High density of macrophages and M2 macrophages for MSC-MEF CM.	Su et al., 2017
PCL-PTHF urethane (P fibers) and PCL-PTHF urethane/collagen I (PC fibers) implanted in defects on the surface of the patellar groove of rat femurs.	After 4 weeks of implantation, newly formed tissues for both P and PC groups with minor inflammatory cells after 4 weeks. Fibrous tissue with a loose and detached for P group; fibrocartilage-like tissue and integration with the surrounding tissue for PC group. After 8 weeks of implantation, hyaline cartilage with round cells in the lacuna for both P and PC groups. More uniform and compact tissue for PC group. Stronger positive immunohistochemical staining of collagen II for PC group after 4 weeks.	Jiang et al., 2018
Random and aligned PCL/Collagen I fibers used to treat full-thickness wounds in diabetic rats.	Remarkable increase of the expression of Arginase I and NOS2 for oriented fibers and consequent stimulation of macrophages transition from M1 to M2. Detection of new blood vessels at the wound site for scaffolds with oriented fibers. Infiltration of fibroblasts and macrophages and collagen deposition in the wound sites for all nanofiber groups.	Sun et al., 2018
Random and aligned PLLA fibers implanted in rats for Achilles tendon repair.	After implantation, for scaffolds with aligned fibers, tendon-like tissue formation, continuous collagen fibers, expression of tendon-specific markers, such as scleraxis, tenomodulin, and Msx-2 (role in preventing tendons from mineralizing). After implantation, for scaffolds with random fibers, substantial chondrogenesis and tissue ossification, high levels of chondro-lineage specific genes, such as collagen type II, Sox9, and aggrecan.	Yin et al., 2015
Polyurethane (PU) grafts with low (void fraction of 53%) and high (void fraction of 80%) porosity, implanted into the infrarenal aorta of rats.	Growth of vimentin-positive fibroblasts, actin-positive myofibroblasts and desmin-positive myocytes at the adventitial interface of the grafts in the early phase after implantation. Growth of myofibroblasts and myocytes within the whole graft wall of the coarse-mesh grafts, 6 months after implantation; while limited cell growth for fine-mesh grafts. Superior cell migration and long-term survival of cells for grafts with high porosity than for grafts with low porosity.	Bergmeister et al., 2013

Another study has investigated how electrospun PCL scaffolds with a novel random-aligned-random structure can be used to mimic bone-ligament connections and native ligaments (Lin et al., 2017). The authors designed a fiber collecting device for the fabrication of electrospun scaffolds with a controlled spatial distribution of random and aligned fibers. The regions of the scaffold with random fibers were then mineralized with Ca-P. *In vitro* tests on human bone marrow MSCs (hBMSCs) revealed that fiber anisotropy modified cells' morphology: polygonal, round-shaped cells without alignment were detected in the random, mineralized regions of the scaffold; while elongated spindle-shaped cells aligned along the fiber direction were visible in the aligned region. Moreover, the aligned fibers significantly up-regulated tendon-specific and tendon-related markers (Tnmd, Mkx) and therefore guided tenogenic phenotypes of hBMSCs; while, the regions with random, mineralized fibers determined the expression of bone-specific markers (Runx-2, Ocn, Opn) and consequently hBMSCs osteogenic phenotypes. Although the authors have not elucidated the underlying cell signaling mechanisms, this work demonstrates that electrospun scaffolds with engineered fiber anisotropy are advantageous to achieve region-specific distribution of tendon- and bone-related genes and find potential application in ligament repair and regeneration of bone-ligament connections.

The possibility to mediate the expression of signaling biomolecules by electrospun fibers and hence guide MSCs differentiation has been demonstrated also by Rahman and co-workers (Rahman et al., 2018). They investigated the odontoblastic differentiation of human dental pulp MSCs (DP-MSCs) on polystyrene (PS) random fibers. The cells cultured on PS mats strongly increased the expression of bone morphogenetic proteins (BMPs) and Wnt ligands that are essential in tooth development: Wnt3a transcript expression was more than 50 folds higher after 4 days of culturing on PS fibers than on standard petri dishes. The levels of odontoblast/osteoblast markers, such as dentin sialophosphoprotein (DSPP), osteocalcin, and bone sialoprotein, were also higher for DP-MSCs cultured on electrospun fibers. The results of this study indicate that nanofibres mimicking the *in vivo* microenvironment are crucial to stimulate the differentiation of DP-MSCs into odontoblasts (specialized cells responsible for dentin formation) by mediating the production of signaling molecules including Wnt3a, and to promote dentinogenesis. Osteogenesis of MSCs has been reported also on random Poly-L-lactic acid (PLLA) fibers, due to cytoskeletal rearrangements and tensions, which in turn influence intracellular mechanotransductive pathways (Yin et al., 2015). In fact, when the cells were treated with Rho kinase (ROCK) inhibitor Y-27632 (inhibitor of myosin-generated cytoskeletal tension), loss of lineage commitment was detected, and cells' morphology was not affected by the fibers topography.

The works here summarized and the others conducted on the interaction of MSCs with electrospun substrates (Tables 1, 2) demonstrate that networks of polymer fibers (random, aligned and hierarchical) are effective in providing topographical and physical cues to guide differentiation of stem cells. These observations have led to the development of bioinspired scaffolds with potential future implications in diverse clinical areas, including the regeneration and repair of bone, tendon, ligament, dentin, and skin.

### Human umbilical vein endothelial cells

Vascular endothelial cells are of fundamental importance for the entire circulatory system, because they are involved in fluid filtration, homeostasis and prevention of thrombosis (Rajendran et al., 2013). Endothelial cells and particularly HUVECs, which are isolated from human umbilical cord veins, are widely used to study cardiovascular diseases and develop biomedical devices for vascular tissue engineering (Lei et al., 2016). One important aspect to consider when designing scaffolds for endothelial cells is the role played by surface topography, at micro- and nano-scale, on cell adhesion, proliferation and migration, to create a physiological environment that stimulates the formation of a functional endothelium.

**Figure 1:**
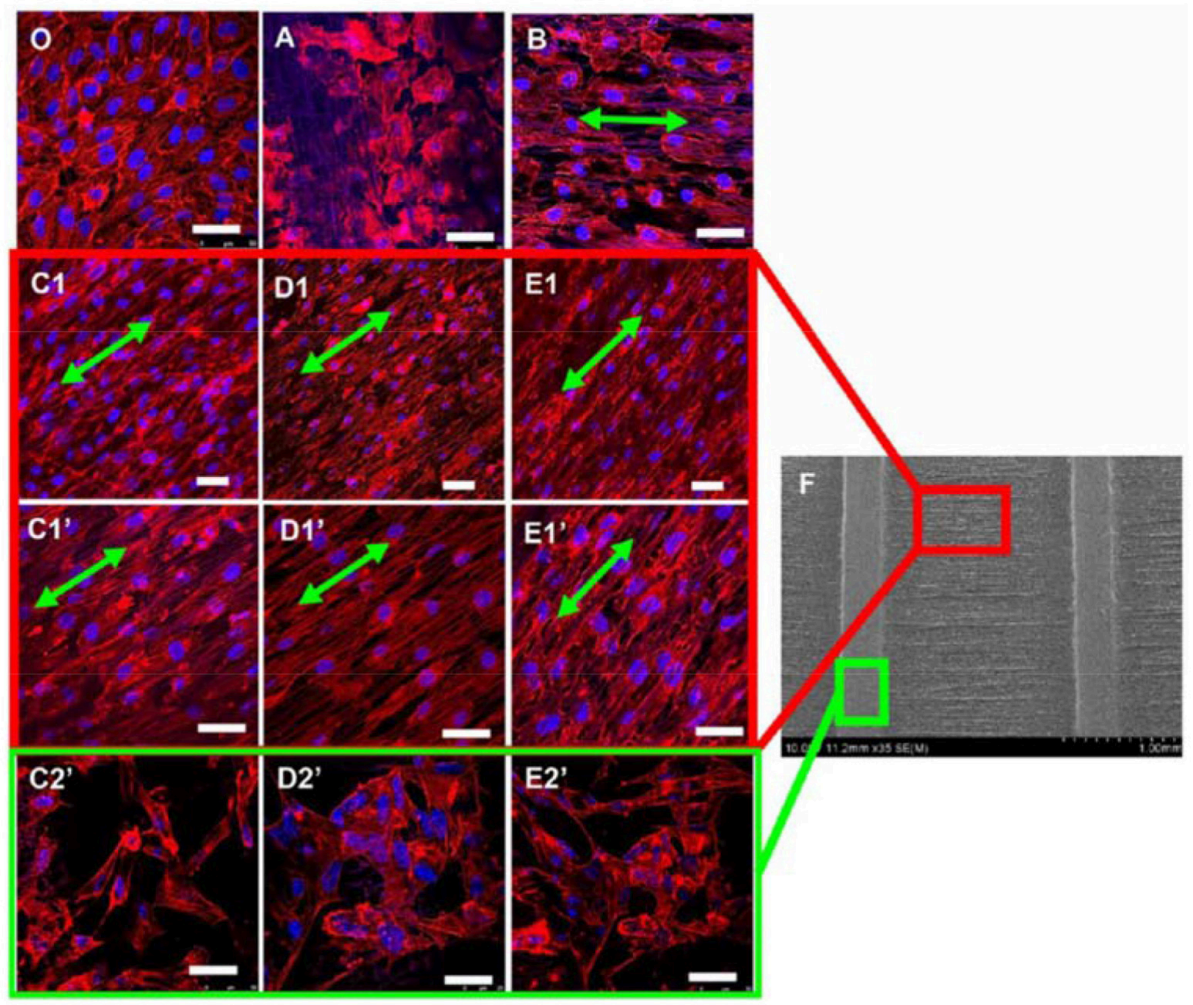
Confocal images of HUVECs (actin filaments in red and nuclei in blue) cultured for 7 days on **(O)** standard petri dish (typical cobblestone-like structure) and electrospun scaffolds with different patterns: **(A)** nonwoven (cells with flat, round shape morphology); **(B)** single directionally aligned pattern (cell alignment along fibers direction, green arrows); **(C1–E1)** anisotropic aligned patterns with interval distances of 300, 800, and 1500 μm, respectively; **(C1**′**-E1**′**)** anisotropic aligned patterns with interval distances of 300, 800, and 1500 μm (high magnification images), respectively (spindle shape along the long fiber axes for cells between the embossments) **(C2**′**- E2**′**)** anisotropic aligned patterns with interval distances of 300, 800, and 1500 μm, respectively (polygonal shape with random stretching for cells on the embossments). **(F)** SEM image of anisotropic aligned pattern. Scale bar = 50 μm. Reprinted with permission from Xu et al. (2015). Copyright 2018 of the American Chemical Society.

A recent work of Ahmed and co-workers has reported on the influence of the diameter of electrospun fibers on HUVECs migration (Ahmed et al., 2018). Aligned poly(lactic-co-glycolic acid) (PLGA) fibers with five different diameters (0.5, 1, 2, 4, and 10 μm) were analyzed. The greater cell displacement in a scratch wound assay was measured for HUVECs seeded on fibers with intermediate diameter (1 and 2 μm) with peak migration velocity of 24 μm/h after 12 h of cell culture. HUVECs were able also to move on scaffolds with 0.5 μm size fibers but at lower migration rates. On these scaffolds cell alignment and polarization, and higher levels of FAK expression were detected. FAK is a non-receptor tyrosine kinase that regulates cell shape, adhesion and motility. The fiber diameter influenced the focal adhesion of HUVECs but not their metabolism or the formation of cell-matrix anchorage points. At 12 h, a significant increase in phosphorylated FAK (pFAK, associated with actin regulation and adhesion dynamics) was detected, which is linked to the peak migration velocity. On the contrary, limited cell motility was observed for scaffolds with 4 and 10 μm fibers. Investigations of the spatial distribution of pFAK revealed that pFAK was localized in the HUVECs cytosol for 0.5, 1.0, and 2.0 μm fibers, and at the cell periphery for 4 and 10 μm fibers (non-uniform distribution). This promoted uniaxial cell morphology and stimulated the migratory process to occur preferentially along the fiber longitudinal direction for scaffolds with intermediate fiber diameter. A similar conclusion has been drawn by other researchers working on HUVECs cultured on micropatterned scaffolds with spatially heterogeneous alignment of poly(D,L-lactide) (PDLLA)/PCL electrospun fibers of 0.5–1 μm size (Xu et al., 2015). Fibrous scaffolds with patterns of random and well-aligned PDLLA/PCL fibers were prepared using a wire spring as template collector. It was observed that the micropatterned scaffolds induced the proliferation of HUVECs and modifications to their cytoskeleton morphology (Figure 1). The lowest values of mean cell body shape index (a parameter indicating the degree of cell polarization) were measured for cells cultured on patterned scaffolds having the longest distance (1,500 μm) between regions with random and aligned fibers, indicating the highest degree of cell polarization and alignment. Furthermore, those scaffolds stimulated the cells to express high levels of angiogenesis-related genes and therefore they have potential applications in vascular tissue engineering. The combination of electrospinning and micro-pattering techniques has proven to be effective for creating hierarchical bio-interfaces that direct the arrangement of endothelial cells and their biological functions (Shin et al., 2017; Yan et al., 2017).

## Computational models

The literature that has been discussed so far in this review provides experimental evidences that the surface topography of biomaterials influences cellular behavior, including cells' alignment, elongation, migration, phenotype transitions and differentiation. *In vitro* and *in vivo* studies are incredibly beneficial to collect data and results on how artificially created micro- and nano-features perform in realistic applications (Tables 1, 2). The underlying mechanisms of cell-material interactions are only partially understood and further investigations are required to define the best scaffold design for promoting the regeneration of a target tissue (Kennedy et al., 2017; Paim et al., 2018). However, time and cost requirements for *in vitro* and *in vivo* tests pose limitations on the use of and reliance on experimental studies alone, together with ethical issues when animal models are concerned. Computational modeling has the significant advantage of facilitating research by conducting thousands of simulated trials with a wide range of variations and for a plethora of complex biological systems (Geris et al., 2018).

**Figure 2:**
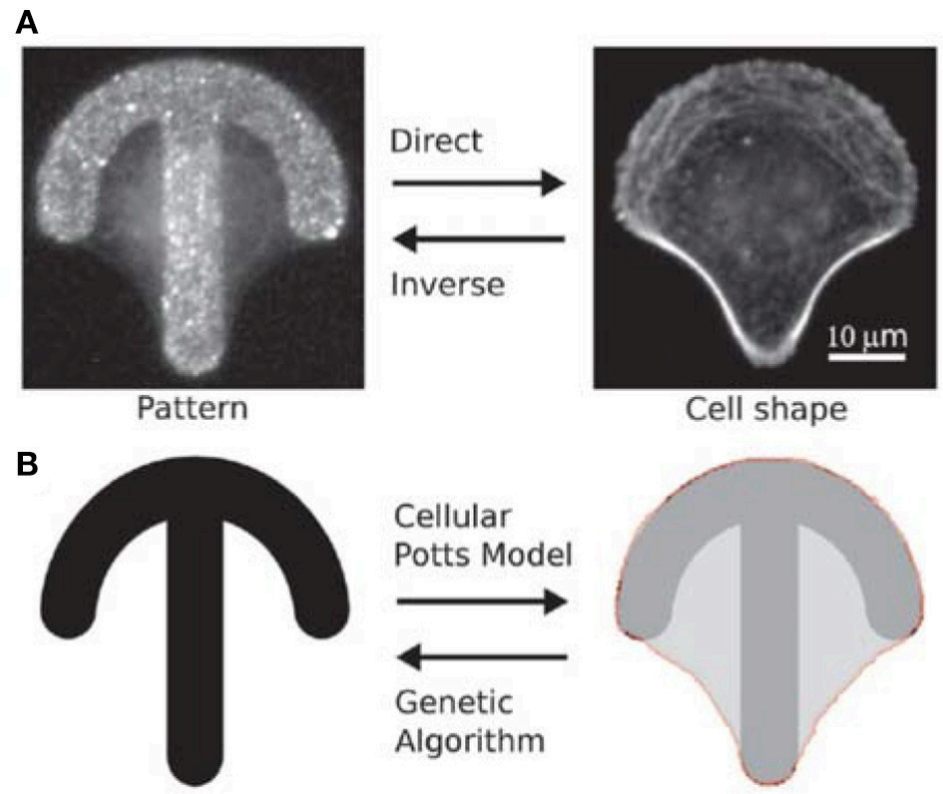
**(A)** Fluorescence images of a HeLa cell stained for actin on a crossbow microstructure coated with fibronectin. Given a micro-pattern, cell shape can be observed with optical microscopy (direct problem). Give a cell shape, it is not always straightforward to experimentally identify the original pattern (inverse problem). **(B)** CPM can be used to predict cell shape on a microstructure, and genetic algorithms can help to define pattern geometry. Reproduced with permission from Albert and Schwarz (2016c). Copyright 2016 of the Royal Society of Chemistry.

Albert and Schwarz have developed mathematical models to predict the dynamics of cell shape and forces on micropatterned substrates (Albert and Schwarz, 2014, 2016a,b). Their models are based on the cellular Potts model (CPM) that allows to simulate the behavior of single or interacting cells by describing them as internally structureless but spatially extended objects on a regular lattice (Voss-Böhme, 2012; Tartarini and Mele, 2015). The number of lattice sites belonging to a single cell defines the area occupied by the cell. By changing the lattice resolution and the indices of the lattice sites, cells with arbitrary shape and shape evolutions can be represented. Initially, the authors compared simulations with experimental data on single cell attached on crossbow, Y and H patterns (Albert and Schwarz, 2014). The model well described how the cell contour adapted to the pattern's geometry and reconstructed the traction forces in agreement with experiments. The forces were higher at the extremities of the patterns (adhesive edges of the contour) and increased with the curvature of the contour depending on the availability of receptors for focal adhesion. The CPM-based model was then used to predict the collective behavior of cells on micropatterns, including cell division, cell-cell contacts and migration (Albert and Schwarz, 2014). The model predicted, for example, that for a cell dividing on a L shaped pattern, the two daughter cells were most likely to be located on the two arms of the L, as confirmed by experimental results. In order to identify the optimal adhesive patterns to control cell functions, CPM was combined with genetic algorithms (GAs) (Albert and Schwarz, 2016c) (Figure 2), which are computational techniques inspired by natural evolution for the heuristic search of problem solutions (McCall, 2005). The migration of cells on rachet micropatterns in a linear arrangement was analyzed and the algorithm predicted that a triangular shape was ideal to guide cell migration in the direction of the tip of the triangle, as also demonstrated experimentally. Differently from what expected though, the most effective pattern to achieve unidirectional migration of cells consisted of asymmetric triangles that were rotated and connected to one another to form a pattern with an almost straight horizontal edge. The computational model developed is a useful tool to predict cell interactions with structured scaffolds and it can be adapted to simulate diverse cellular processes.

With a distinct lack of literature on computational/numerical modeling that predicts how cells interact with electrospun nanofibrous structures (role of roughness and topography), there is a clear gap in the field which has great potential if correctly pursued. This will open even more possibilities to design and create novel fibrous scaffolds with engineered surface structures (Ziebert and Aranson, 2016). For example, computer aided characterization of complex biointerfacial interactions of specific polymer fibers could be created. Computational algorithms and numerical solutions could be formulated to generate a method of predicting the most suitable surface topography of electrospun mats for specific cells and to prompt tissue regeneration processes. In the development of computational models that describe how cell behavior is affected by the surface properties of electrospun scaffolds, geometrical parameters to be considered include fibers diameter, fibers organization and degree of alignment, porosity of the mat, presence of nanostructures or nanopores on single fiber surface, overall roughness of the electrospun mat. All these aspects have been evaluated experimentally, as discussed in the previous section of this mini review.

## Conclusions

Electrospun nanofibers have become vital structures for a plethora of different applications, with the field of biomedical engineering being, arguably, one of the most important. Thanks to the versatility of electrospinning, nanofibrous scaffolds can be tailored and modified to improve their biocompatibility for applications such as tissue engineering, drug delivery and wound dressings. For example, electrospun mats have been used in clinical studies for the treatment of arterial occlusive disease, skin cancer and diabetic foot (Table 3). As discussed in this review, fiber alignment, micropatterning, and controlled porosity of nanofibrous mats have all been found to have significant effect on cellular behavior, inducing cell attachment, migration and differentiation. Extensive research has been conducted on exploring morphological cues provided by 2D electrospun mats, and only recently fibrous 3D scaffolds have been proposed to closely mimic the ECM structure (Cai et al., 2013; Lee et al., 2014; Cho et al., 2016; Hwang et al., 2018; Unnithan et al., 2018). The studies conducted so far have demonstrated that a fine tuning of the 3D porosity of the electrospun scaffolds is crucial to promote cell infiltration. Future research in the field should combine experimental studies with numerical and computational modeling for the design and fabrication of novel micro- and nanostructured 3D scaffolds. Computer aided simulations could not only be used to predict cell interaction with specific topography but be formulated in a manner which then advises on the most suitable functional group (or biological molecule) that ought to be immobilized on the surface or embedded within the scaffold. This would require taking in consideration a complex combination of parameters that include the chemical composition of the scaffold (exposed chemical groups, wetting properties, and biodegradation), micro- and nano-porosity, organization of the fibrous network (random or aligned fibers) and mechanical properties of the scaffold.

**Table 3:** Clinical trials of electrospun scaffolds.

**Study**	**Status**	**Condition/disease**	**Aim**	**Number of participants**	**Scaffold**	**Results**
Experimental study of the vascular prosthesis manufactured by electrospinning (NCT02255188)	Completed	Arterial occlusive disease	Determination of the safety of electrospun vascular grafts for the development of thrombosis.	120	PCL grafts; PCL/gelatin grafts; PLGA/PCL/gelatin grafts; Nylon 6 grafts.	Not currently available
EktoTherix™ regenerative tissue scaffold for repair of surgical excision wounds (NCT02409628)	Completed	Non-melanoma skin cancer; Basal cell carcinoma; Squamous cell carcinoma	Assessment of the safety and performance of EktoTherix™ Tissue Repair Scaffold for the treatment of full-thickness, dermatologic wounds due to the surgical removal of non-melanoma skin cancers.	12	EktoTherix™ Tissue Repair Scaffold:	Not currently available
Clinical trial for the treatment of diabetic foot ulcers using a nitric oxide releasing patch: PATHON	Completed	Diabetic foot	Evaluation of the effectiveness and safety of nitric oxide releasing wound dressings for the treatment of diabetic foot ulcers.	100	Multilayer polymeric transdermal patch with a continuous release of nitric oxide (polyurethane-based fibers).	Not currently available
Controlled nitric oxide releasing patch vs. meglumine antimoniate in the treatment of cutaneous Leishmaniasis	Terminated	Cutaneous Leishmaniasis	Evaluation of the effectiveness of a nitric oxide topical donor for the treatment of cutaneous leishmaniasis.	178	Multilayer polymeric transdermal patch with a continuous release of nitric oxide (polyurethane-based fibers).	Not currently available

*The data are obtained from ClinicalTrials.gov, a resource provided by the U.S. National Library of Medicine (Accessed on September 2018)*.

## Author contributions

AD and EM contributed to the conception and design of the study. AD and EM wrote the first draft of the manuscript. DT wrote sections of the manuscript. All authors contributed to manuscript revision, read and approved the submitted version.

### Conflict of interest statement

The authors declare that the research was conducted in the absence of any commercial or financial relationships that could be construed as a potential conflict of interest.
